# Rescue of a panel of Hemophilia A-causing 5’ss splicing mutations by unique Exon-specific U1snRNA variants

**DOI:** 10.1186/s10020-025-01176-8

**Published:** 2025-03-27

**Authors:** Laura Peretto, Claudia D’angiolillo, Paolo Ferraresi, Dario Balestra, Mirko Pinotti

**Affiliations:** Department of Life Sciences and Biotechnology, University of Ferrara, Ferrara, 44121 Italy

**Keywords:** Hemophilia A, F8 gene, Splicing mutations, U1snRNA, Exon skipping, RNA therapeutics, Coagulation factor VIII, Gene therapy, ExSpeU1, Engineered U1snRNA

## Abstract

**Background:**

Aberrant mRNA splicing is a well-established pathogenic mechanism for human disease, but its real impact is hardly predictable and underestimated. Splicing can be therefore modulated for therapeutic purposes, and splicing-switching molecules are in clinics for some diseases. Here, conscious that over 10% of all pathogenic mutations occurs at 5’ss, we aimed at characterizing and rescuing nine 5’ss mutations in three models of defective *F*8 exons whose skipping would lead to factor VIII (FVIII) deficiency (Hemophilia A), the most frequent coagulation factor disorder.

**Methods:**

HEK293T cells were transfected with *F8* minigene variants, alone or with engineered U1 small nuclear RNAs (U1snRNAs), and splicing patterns analysed via RT-PCR.

**Results:**

All 5’ss mutations induced exon skipping, and the proportion of correct transcripts, not predictable by computational analysis, was consistent with residual FVIII levels in patients. For each exon we identified a unique engineered U1snRNAs, either compensatory or Exon Specific (ExSpeU1), able to rescue all mutations. Overall, ExSpeU1s were more effective than compensatory U1snRNAs, particularly in the defective exons 6 and 22.

**Conclusions:**

Data highlight the importance of splicing assays to elucidate genotype-phenotype relationships and proved the correction efficacy of ExSpeU1s for each targeted defective *F8* exon, thus expanding their translational potential for HA.

**Supplementary Information:**

The online version contains supplementary material available at 10.1186/s10020-025-01176-8.

## Background

Gene expression in higher organisms greatly relies on the splicing process implying the precise definition of exons and removal of introns from precursor messenger RNA (pre-mRNA), and virtually all mRNAs in mammals are regulated by alternative splicing (Nilsen and Graveley 2010; Wang et al. 2008). Its complexity, involving the very large spliceosome machinery and several *cis* regulatory elements, makes it susceptible to derangements, and, not surprisingly, aberrant splicing represents the pathogenic mechanism for a relevant proportion of human disease-causing mutations, particularly of severe forms (http://www.hgmd.org/). However, the relevance of splicing in disease is largely underestimated since many exonic variations can impair it (Lombardi et al. 2021; Sterne-Weiler et al. 2014). This occurs because the amino acid code overlaps with the intricate network of splicing regulatory elements that govern the splicing process (Falanga et al. 2014; Soemedi et al. 2017).

In this context, many efforts have been made to develop splicing switching-molecules for therapeutic purposes, which has led to the development of antisense oligonucleotides that induce exon-skipping in the *DMD* gene and that are currently in clinical trials for Duchenne Muscular Dystrophy (Servais et al. 2022) or promote *SMN2* exon 7 inclusion, in clinical practice for patients with Spinal Muscular Atrophy (Finkel et al. 2017). On the other hand, exon skipping, the most common event caused by splicing changes, can be counteracted by improving exon definition through the small nuclear ribonucleoprotein U1snRNP. In the earliest splicing step, the U1snRNP plays a key role in exon definition by binding to the 5’ splice site (5’ss) through base-pair complementarity with its RNA component (U1snRNA) (Conti et al. 2013; Roca et al. 2013). We and others have demonstrated that U1snRNA variants engineered to strengthen complementarity with the 5’ss (compensatory U1snRNA) or targeting intronic sequences downstream of the defective exon (Exon Specific U1snRNAs; ExSpeU1) can rescue exon-skipping variants, and their efficacy has been proven in several cellular and animal models of human disease (Fernandez Alanis et al. 2012; Rogalska et al. 2016; Sacchetto et al. 2021; Peretto et al. 2023; Balestra et al. 2019, 2020a, b). Notably, they effectively rescue exon-skipping variants occurring at the 5’ss, 3’ss or within the exon (Lombardi et al. 2021; Zhang et al. 2024).

Conscious that over 10% of all pathogenic mutations occur at splice sites, particularly at the 5’ss (Krawczak et al. 2007; López-Bigas et al. 2005), in this study, we challenged engineered U1snRNAs on nine 5’ss changes in three models of defective *F*8 exons whose skipping would lead to coagulation factor VIII (FVIII) deficiency (Hemophilia A; HA, OMIM:306700), the most frequent coagulation factor disorder (Peyvandi et al. 2016). Through the expression of *F8* minigenes we dissected their differential causative role, which was not predicted by computational tools, and demonstrated that the exon skipping mechanism can be efficiently counteracted by a unique engineered U1snRNA for the specific exon. Moreover, we show for the first time that, for some variants, ExSpeU1 is more efficient than its compensatory counterpart, which further increases its therapeutic potential.

## Methods

### Computational analysis

The computational prediction of 3’ and 5’ splice sites strength was conducted by using the SpliceRover (http://bioit2.irc.ugent.be/rover/splicerover) tool.

MaxEntScan (http://hollywood.mit.edu/burgelab/maxent/Xmaxentscan_scoreseq.html), and the RNAcofold (http://rna.tbi.univie.ac.at/cgi-bin/RNAWebSuite/RNAcofold.cgi) tools, with the available Maximum Entropy Model (MEM), Maximum Dependence Decomposition Model (MDMM), First-order Markov Model (MM), Weight Matrix Model (WMM), and Minimum Free Energy (MFE), respectively, were used to bioinformatically predict the 5’ss score in normal and muted conditions. All data are reported in Supplementary Table 1.

### Creation of recombinant plasmids

To create minigenes, the selected human F8 genomic regions (NC_000023.11) were amplified from the genomic DNA of a healthy subject using high-fidelity Q5^®^ DNA-Polymerase (New England Biolabs, Ipswich, MA, USA), and subsequently cloned into the pTB plasmid (Baralle et al. 2003) by exploiting the NEBridge golden-gate assembly using BsmbI-v2 (New England Biolabs, Ipswich, MA, USA).

The pTB-F8 IVS11 (c.1752) includes the last 442 bp of intron 10, exon 11 (215 bp), and the first 236 bp of intron 11. The pTB-F8 IVS6 (c.787) includes the last 469 bp of intron 5, exon 6 (117 bp), and the first 454 bp of intron 6. The pTB-F8 IVS22 (c.6429) includes the last 451 bp of intron 21, exon 22 (156 bp), and the first 433 bp of intron 22.

The F8 variants (c.787 + 2T > C; c.787 + 3 A > G; c.787 + 3 A > T; c.787 + 5G > A; c.787 + 6T > C; c.1752 + 5G > T; c.1752 + 5G > C; c.1752 + 5G > A; c.6429 + 5G > T) were introduced by site-directed mutagenesis. Expression vectors for the U1snRNA variants, either designed to bind to the mutated 5’ss (compensatory U1snRNA; U1 comp) or to downstream intronic sequences (Exon Specific U1snRNA, ExSpeU1snRNA) were created as previously reported (Balestra et al. 2015). All the vectors were validated by sequencing. Sequences of oligonucleotides are provided in Supplementary Table 2.

### Expression in mammalian cells and mRNA studies

Human Embryonic Kidney (HEK293T) cells (Pignani et al. 2018) were seeded in 12-well plates and transiently transfected with 500ng of pTB-F8 variants and 1.5X molar amounts of engineered U1snRNA (Lombardi et al. 2021; Balestra et al. 2019) using Lipofectamine™ 2000 reagent (ThermoFisher Scientific, Waltham, MA, USA), as indicated by the manufacturer’s instructions.

Twenty-four hours post-transfection the total RNA was isolated with TRIzol Reagent^®^ (ThermoFisher Scientific, Waltham, MA, USA), reverse transcribed with random primers with M-MLV reverse transcriptase (RT)(Thermo Fisher Scientific, Waltham, MA, USA), and amplified with the primers Alpha globin F, and Bra2 Rev oligonucleotides designed on the upstream and downstream pTB exons, respectively. The PCR was run for 40 cycles at the following conditions: 30 s at 95 °C, 30 s at 53 °C, and 50 s at 72 °C, and the amplicons were resolved on 2% agarose gel. Densitometric analysis for the quantification of correct and aberrant transcripts was performed using the ImageJ software (https://imagej.net). The Student’s t-test was used for statistical analysis with *p* > 0.05 considered not significant (ns).

## Results and discussion

Variants at the 5’ss account for approximately 10% of all the unique 3052 *F8* variants reported in the *F8* database (https://f8-db.eahad.org/, May 2024) and, with the exclusion of the severest ones occurring at the conserved dinucleotide + 1G + 2T, are associated with variable degrees of HA severity.

In this study we selected three model *F8* exons (exons 6, 11 and 22) that the bioinformatic analysis predicted to be well defined, as indicated by the high 5’ss and 3’ss scores (5’ss 89, 98 and 91, 3’ss 99, 50 and 99 for exons 6, 11 and 22, respectively) (Table 1). The prediction did not foresee adjacent strong cryptic 5’ss, which points toward an exon skipping event in the presence of changes reducing exon definition.

**Table 1:** Reporting the mean characteristics of the selected *F8* mutation. Patients data are from the Coagulation Factor Variant Databases (https://dbs.eahad.org/FVIII, accessed on 1/08/2024). 5’ss and 3’ss scores predicted through the SpliceRover tool (http://bioit2.irc.ugent.be/rover/splicerover) of *F8* exons 6, 11, and 22. n.r: not reported

EXON	3’ss score	5’ss score	*N* mutations	*N* Patients	Mutations	*N* patients	FVIII: C range (%)	FVIII: C median (%)	Severity
**6**	0,99	0,89	7	33	c.787 + 2T > C	2	< 1–3.3	1,9	Moderate
					c.787 + 3 A > G	15	< 1–6	2,5	Moderate
					c.787 + 3 A > T	3	8–10	10	Mild
					c.787 + 5G > A	3	2	2	Moderate
					c.787 + 6T > C	3	12	12	Mild
**11**	0,5	0,98	5	11	c.1752 + 5G > T	1	n. r.	n. r.	n. r.
					c.1752 + 5G > C	1	2	2	Moderate
					c.1752 + 5G > A	5	7–20	10,5	Mild
**22**	0,99	0,91	5	13	c.6429 + 5G > T	3	n. r.	n. r.	n. r.

Among all variants occurring at the exons 6, 11 and 22 5’ss, we selected those that, based on previous studies (Peretto et al. 2023; Scalet et al. 2019), are potentially rescuable by the U1snRNA-mediated approach (+ 2T > C and those located at positions + 3 to + 6 within the 5’ss), and are associated with different degrees of HA severity (Table 1).

**Fig. 1:**
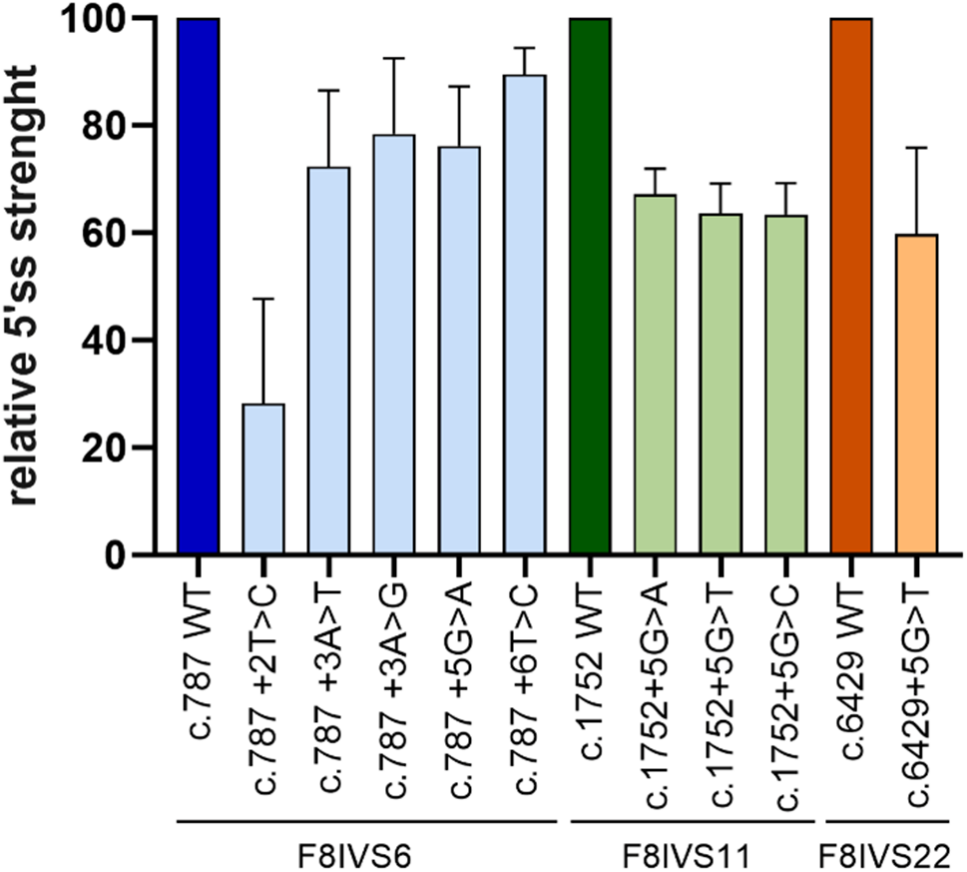
Selected 5’ss mutations and predicted 5’ss strength. Bar plot representing the mean ± standard deviation (SD) of all 5’ss score predictions calculated by SpliceRover, MFE, MAXENT, MDD, MM, WMM tools expressed as percentages. The score of natural 5’ss was set to 100%. See supplementary Table 1 for raw data and calculations

We exploited different splicing algorithms to predict the impact of the selected variants on the 5’ss score and found that they lead to a general decrease in the 5’ss strength, with the c.787 + 2T > C and the c.787 + 6T > C changes predicted to be the most and the least detrimental, respectively. On the other hand, all the others were predicted to have a comparable effect (Fig. 1; suppl Table 1).

**Fig. 2:**
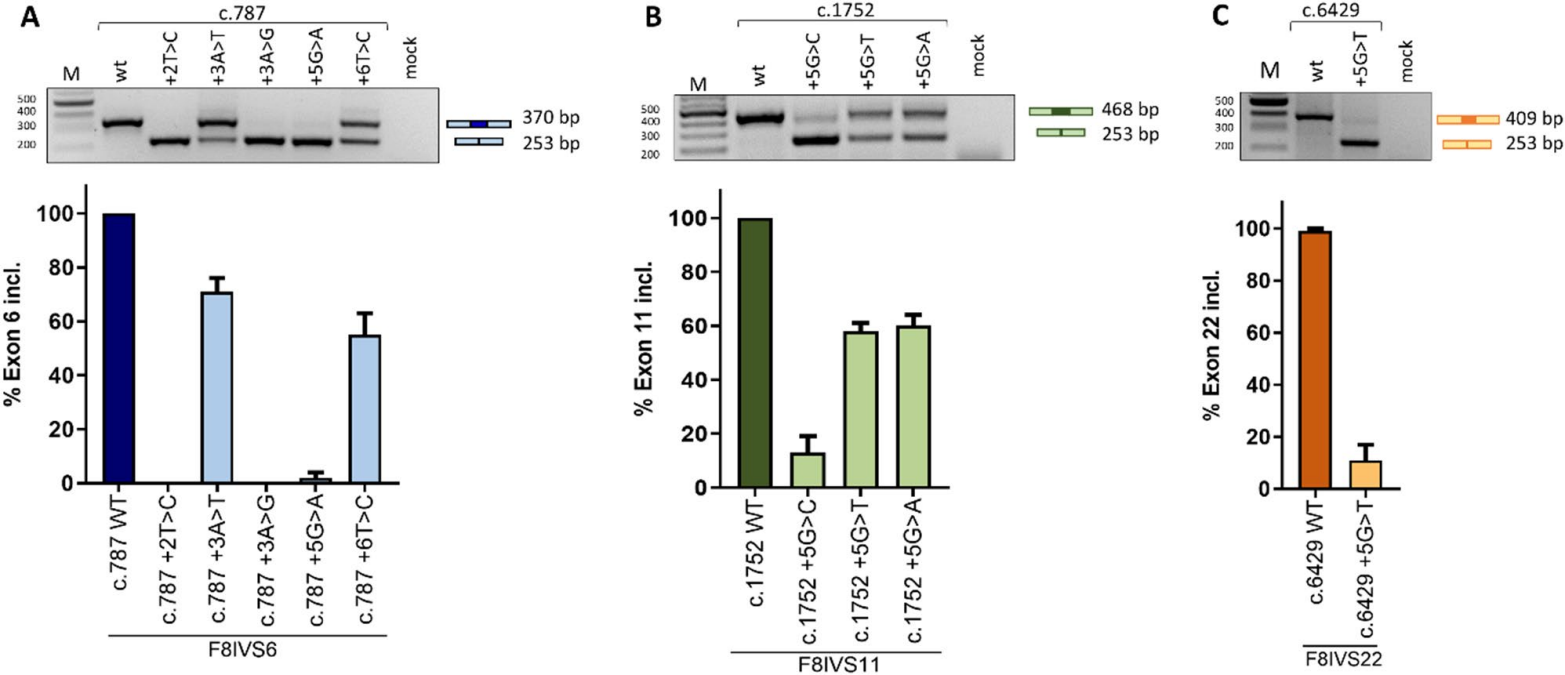
Splicing mutations mainly induce exon skipping. **(A-B-C)** Splicing pattern of the wild type and mutant pTB-F8 minigenes for *F8* exons 6, 11 and 22. The upper panels show the amplified products separated on 2% agarose gel. The schematic representation of transcripts, and size, is reported on the right of each panel. Size of the expected transcripts is reported. The bar plots are reporting the densitometric quantification of the percentage of exon inclusion, calculated with ImageJ software and expressed as mean ± standard deviation (SD) from three independent experiments. M: molecular 1 Kb marker. Mock: untransfected cells

To experimentally investigate splicing patterns, we exploited minigenes including the affected *F8* exon and the surrounding introns in the well-established exon trapping plasmid pTB (Fernandez Alanis et al. 2012). The expression of the wild type minigenes experimentally demonstrated that all three exons are well defined, as indicated by the presence of correctly spliced transcripts (Fig. 2), which is consistent with the prediction of strong 3’ss and 5’ss. Conversely, all variants induced exon skipping, albeit to different extents. Skipping of exons 6 and 22 would maintain the frame of the resulting FVIII exon-skipped transcripts, but without part of the A1 and C1 domains, thus lacking an important glycosylation site (Ito et al. 2022) or membrane-binding loop (Childers et al. 2023), respectively. On the other hand, exon 11 skipping results in a frameshift and thus the insertion of a premature stop codon (c.1579), which predicts the synthesis of a truncated FVIII isoform. Therefore, in patients, the residual FVIII activity associated with *F8* variants would depend on the residual amount of correctly spliced transcripts. Densitometric analysis of the bands led us to estimate the relative proportion of correctly spliced transcripts. The c.787 + 2T > C, c.787 + 3 A > G and c.787 + 5G > A changes led to almost complete skipping of exon 6, whereas the c.787 + 3 A > T and c.787 + 6T > C changes were compatible with appreciable levels of correctly spliced transcripts (71% and 55%, respectively) (Fig. 2A). The c.1752 + 5G > A and c.1752 + 5G > T variants in exon 11 were associated with 58% and 64% correct transcripts, whereas the c.1752 + 5G > C variant markedly impaired exon inclusion (13% exon inclusion) (Fig. 2B). In the model of exon 22, the c.6429 + 5G > T change markedly impaired splicing, with only 13% of transcripts correctly spliced (Fig. 2C). Notably, variants occurring at the same positions (c.787 + 3 A > G, c.787 + 3 A > T and c.1752 + 5G > A, c.1752 + 5G > T, c.1752 + 5G > C), despite being predicted by different algorithms to impair exon definition to a similar extent, revealed different correctly spliced levels. This observation further highlights the importance of experimental evaluation to assess the pathogenic impact of variants, which is hardly predictable by bioinformatics tools.

This semi-quantitative assessment shed light on genotype-phenotype relationships. The c.787 + 2T > C, c.787 + 3 A > G and c.787 + 5G > A changes in exon 6, the c.1752 + 5G > C in exon 11 and the c.6429 + 5G > T in exon 22 were associated with remarkably impaired splicing in accordance with the very reduced FVIII levels and a moderate phenotype (Table 1). The remaining detrimental impact was modest, in accordance with a mild phenotype and appreciable FVIII levels.

Overall, expression studies demonstrated the impact of 5’ss variants on splicing processing and provided models, particularly the severest ones, to evaluate the U1snRNA-mediated correction approach, which has been effective in several cellular and in vivo disease contexts (Romano et al. 2022; Donegà et al. 2020; Lee et al. 2019; Donadon et al. 2019). Engineered U1snRNAs can be delivered *via* viral vectors as a personalized gene therapy with one intervention only, have the advantage of maintaining proper transcriptional control in the physiological tissue only, and the proof-of-principle for their efficacy has been provided in mouse models of Spinal Muscular Atrophy (Donadon et al. 2019) and Familial Dysautonomia (Donadon et al. 2018). Noticeably, it has been shown that even a single copy of engineered U1snRNA integrated into the genome is enough to ensure rescue both in cellular and animal models (Dal Mas et al. 2015).

**Fig. 3:**
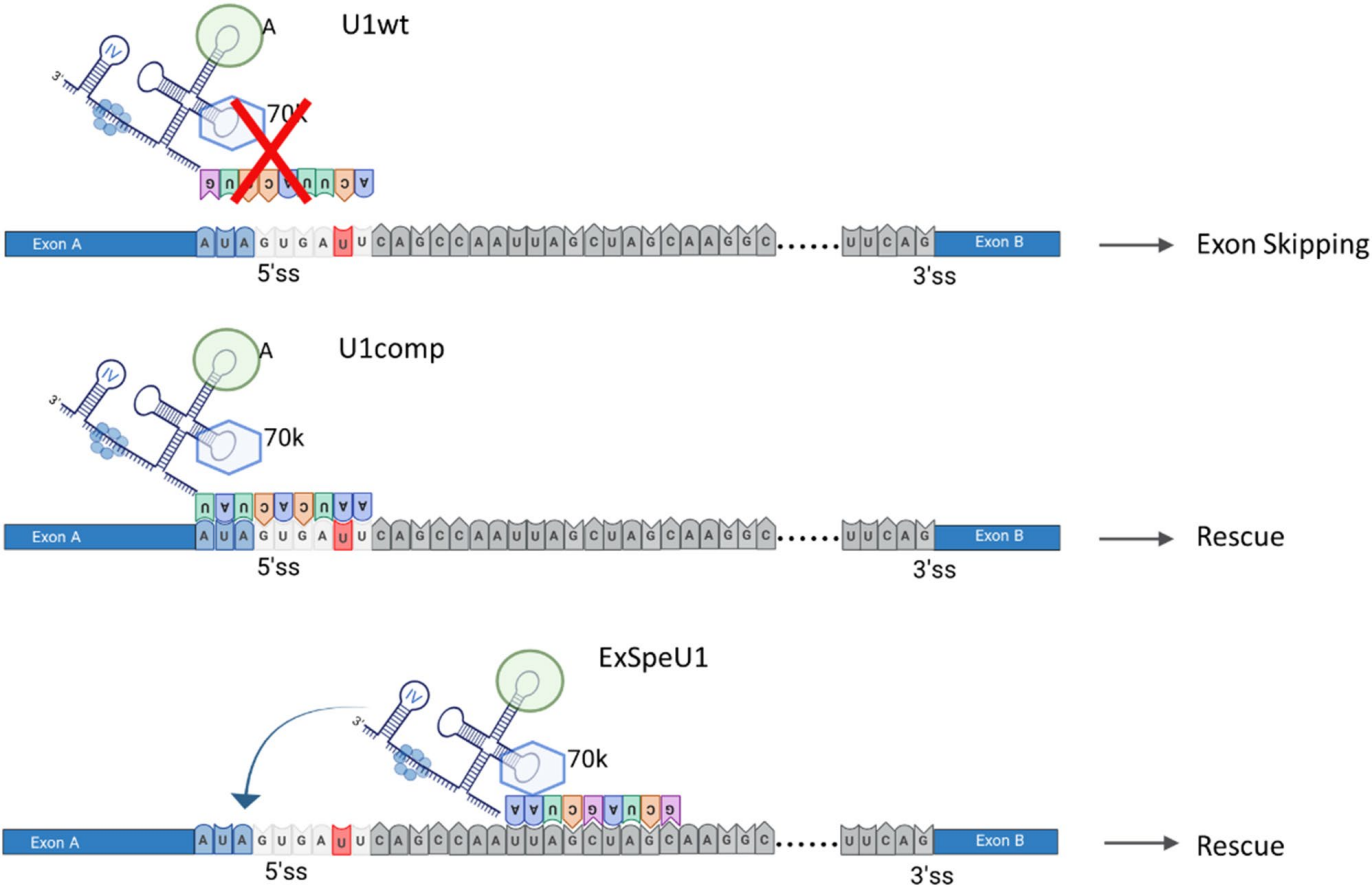
Molecular mechanism of U1comp and ExSpeU1. Schematic representation of the molecular mechanisms of U1comp and ExSpeU1. (Upper) The splicing variant occurring at the 5’ss (here + 5G > U) impairs recognition by the endogenous U1snRNA (U1wt), thus leading to exon skipping. (Middle) The compensatory U1snRNA (U1comp) perfectly binds with the mutated 5’ss, thus restoring spliceosome assembly and rescuing splicing. (Lower) ExSpeU1, by binding on downstream intronic sequences, promotes exon and intron definition (arrow) by its 70k and SL4 elements through interaction with other context-specific splicing factors. The 5’ss is reported in light blue (the last three nucleotides of exon) and light grey. The splicing mutation is reported in red. Exons and intron are in light blue and grey, respectively

On the other hand, for Hemophilia A as well as the majority of human genetic disorders, the heterogeneous mutational pattern makes designing a single therapeutic U1snRNA for each variant difficult, which led us to expand its applicability by developing a second generation called ExSpeU1. In fact, by targeting intronic sequences downstream the defective exon, we have proven the ability, with a single ExSpeU1, to rescue splicing patterns in the presence of multiple changes at splice sites or within the exon (Lombardi et al. 2021; Fernandez Alanis et al. 2012). Moreover, by targeting gene-specific intronic regions, they have the potential for increased specificity and minor risk for off-target effects, as demonstrated in different mouse models of diseases (Rogalska et al. 2016; Balestra et al. 2020a, b). Experimental data showed that the two generation of U1snRNA variants act through different mechanisms (Fig. 3). While the compensatory U1snRNA directly binds to the mutated 5’ss and therefore restores spliceosome assembly on the mutated 5’ss, making their design rather straightforward, the ExSpeU1s ensure that two important U1snRNA elements (70 K and SL4) are loaded on pre-mRNA in the proximity of 5’ss and promoting intron and exon definition, respectively. Noticeably, ExSpeU1s do not act as antisense molecules by targeting intronic sequences with silencer function and don’t require the endogenous U1snRNA (Rogalska et al. 2016).

**Fig. 4:**
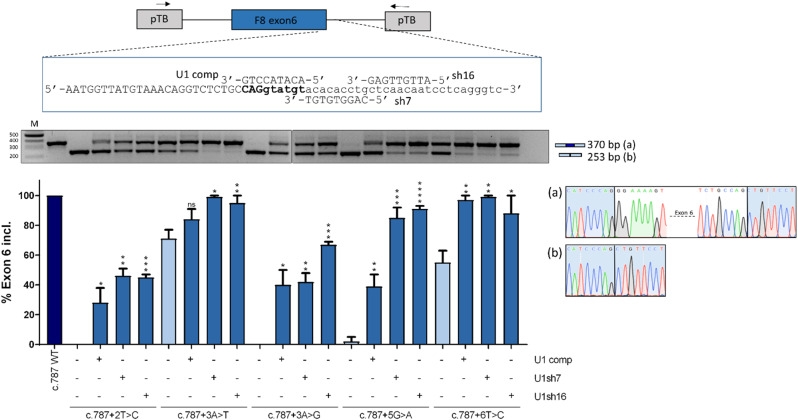
Rescue of *F8* exon 6 with engineered U1snRNAs. The schematic representation of the pTB-F8IVS6 minigene is reported in the upper panel. Arrows represent primers used for RT-PCR amplification. Exonic and intronic sequences are in upper and lower case, respectively, and the donor site is in bold. The modified 5’ tail of each engineered U1snRNA is aligned with the target-binding sequences on the pre-mRNA. The splicing pattern was evaluated by RT-PCR, and amplified products were resolved on a 2% agarose gel. The schematic representation of transcripts, with the expected size, is reported on the right. Electropherograms of transcripts is reported on the right. Densitometric quantification of the bands was calculated with ImageJ software and reported in the lower panel. The bar plot represents the percentage of *F8* exon 6 inclusion expressed as mean ± standard deviation (SD) from three independent experiments. The wild type (WT, dark blue) and mutant (c.787 + 2T > C/+3 A > T/+3 A > G/+5G > A/+6T > C, light blue) minigenes have been transfected in HEK293T cells alone, or co-transfected with plasmids encoding for the engineered U1snRNAs (blue). The Student’s t-test was used for statistical analysis, with *p* > 0.05 considered not significant (ns); *p* < 0.05*; *p* < 0.01**; *p* < 0.001***; *p* < 0.0001****. M: 1 Kb molecular marker

**Fig. 5:**
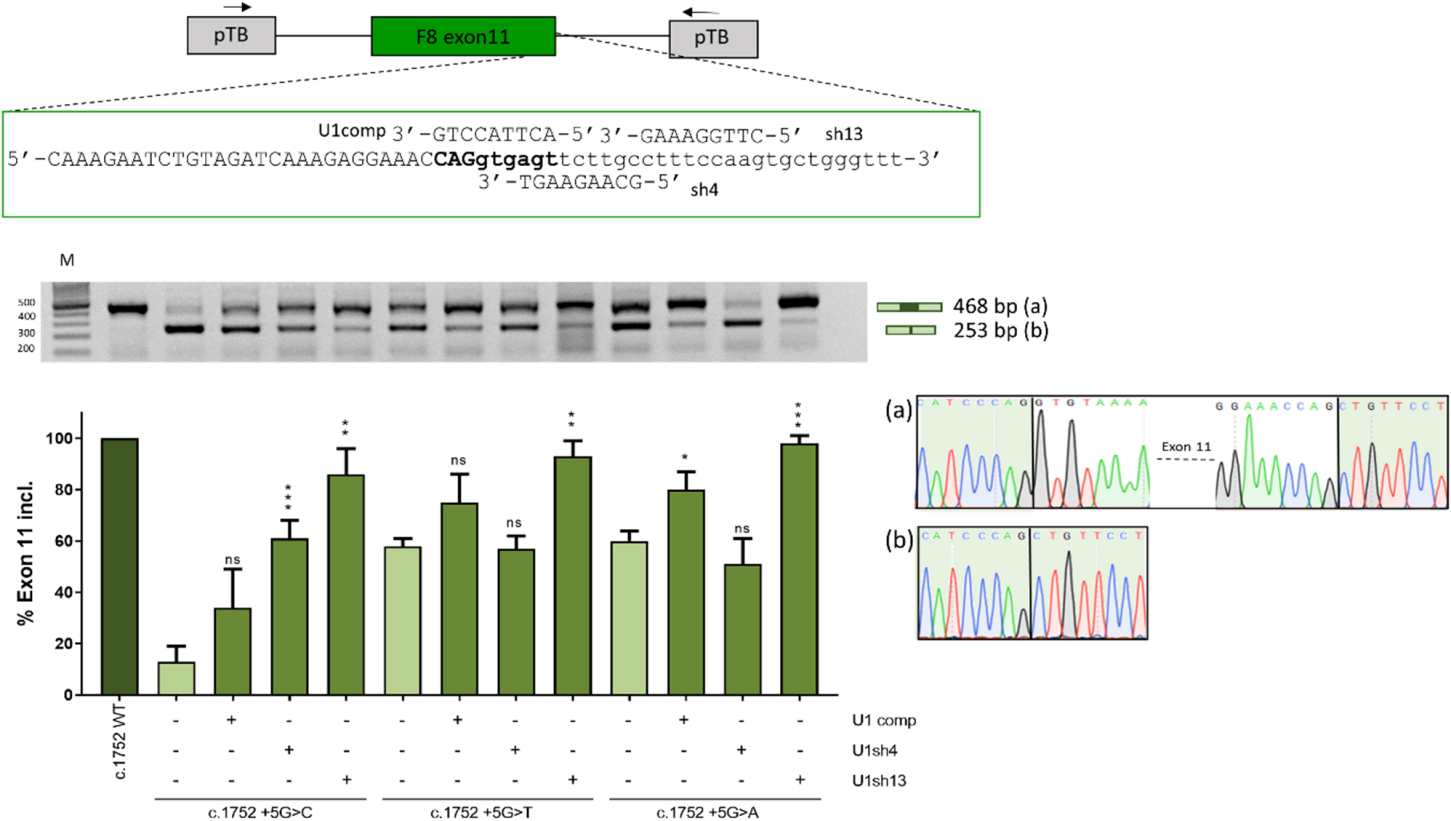
Rescue of *F8* exon 11 with engineered U1snRNAs. The schematic representation of the pTB-F8IVS11 minigene is reported in the upper panel. Arrows represent primers used for RT-PCR amplification. Exonic and intronic sequences are in upper and lower case, respectively, and the donor site is in bold. The modified 5’ tail of each engineered U1snRNA is aligned with the target-binding sequences on the pre-mRNA. The splicing pattern was evaluated by RT-PCR, and amplified products were resolved on a 2% agarose gel. The schematic representation of transcripts, with the expected size, is reported on the right. Electropherograms of transcripts is reported on the right. Densitometric quantification of the bands was calculated with ImageJ software and reported in the lower panel. The bar plot represents the percentage of *F8* exon 11 inclusion expressed as mean ± standard deviation (SD) from three independent experiments. The wild type (WT, dark green) and mutant (c.1752 + 5G > C/+5G > T/+5G > A, light green) minigenes have been transfected in HEK293T cells alone, or co-transfected with plasmids encoding for the engineered U1snRNAs (green). The Student’s t-test was used for statistical analysis, with *p* > 0.05 considered not significant (ns); *p* < 0.05*; *p* < 0.01**; *p* < 0.001***; *p* < 0.0001****. M: 1 Kb molecular marker

**Fig. 6:**
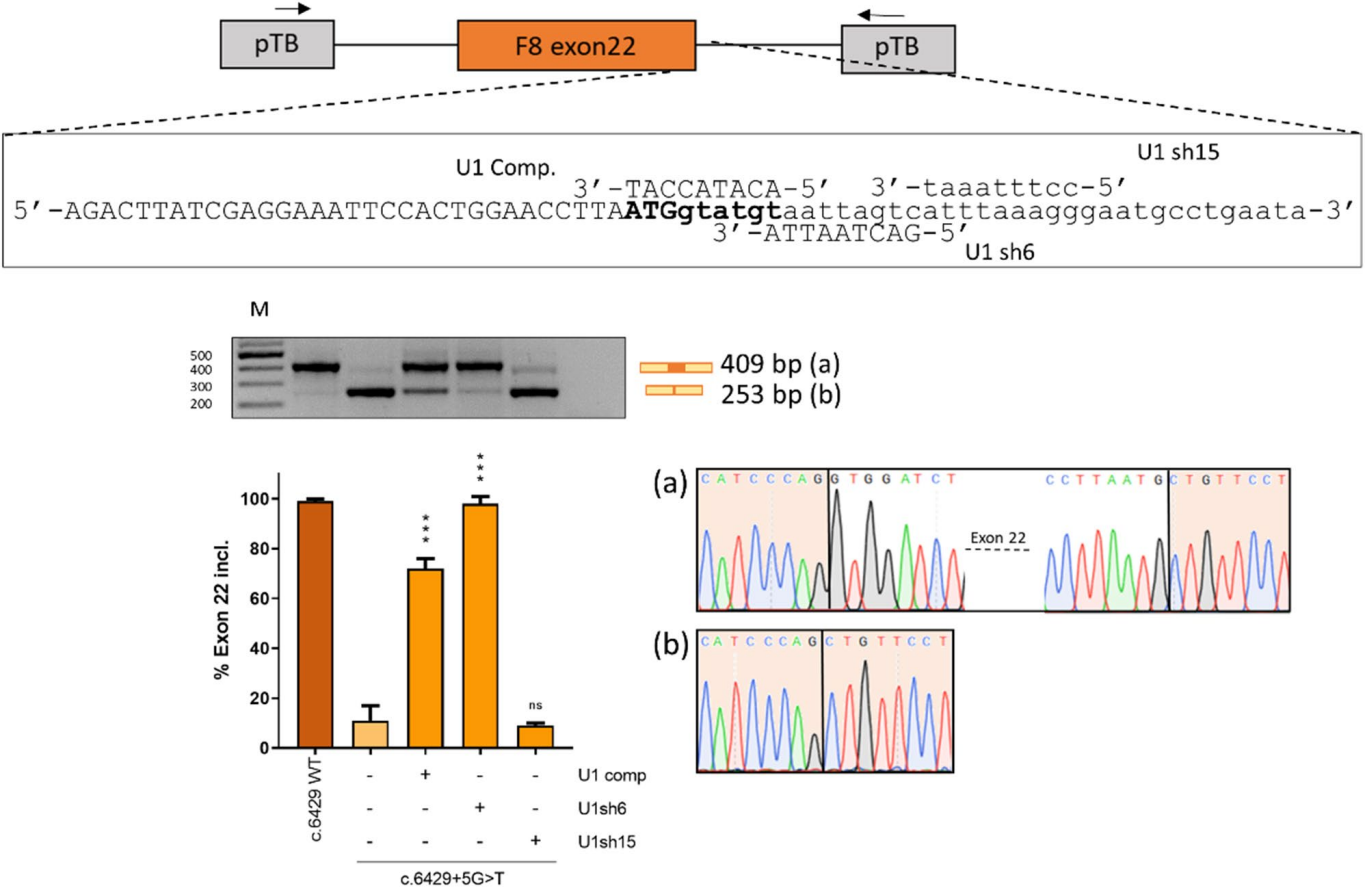
Rescue of *F8* exon 22 with engineered U1snRNAs. The schematic representation of the pTB-F8IVS22 minigene is reported in the upper panel. Arrows represent primers used for RT-PCR amplification. Exonic and intronic sequences are in upper and lower case, respectively, and the donor site is in bold. The modified 5’ tail of each engineered U1snRNA is aligned with the target-binding sequences on the pre-mRNA. The splicing pattern was evaluated by RT-PCR, and amplified products were resolved on a 2% agarose gel. The schematic representation of transcripts, with the expected size, is reported on the right. Electropherograms of transcripts is reported on the right. Densitometric quantification of the bands was calculated with ImageJ software and reported in the lower panel. The bar plot represents the percentage of *F8* exon 22 inclusion expressed as mean ± standard deviation (SD) from three independent experiments. The wild type (WT, dark orange) and mutant (c.6429 + 5G > T, light orange) minigenes have been transfected in HEK293T cells alone, or co-transfected with plasmids encoding for the engineered U1snRNAs (orange). The Student’s t-test was used for statistical analysis, with *p* > 0.05 considered not significant (ns); *p* < 0.05*; *p* < 0.01**; *p* < 0.001***; *p* < 0.0001****. M: 1 Kb molecular marker

Therefore, for each target exon, we designed a compensatory U1snRNA (U1comp) fully complementary to the wild type 5’ss and two ExSpeU1snRNAs (U1sh), which target downstream intronic sequences. Co-transfection experiments (Figs. 4, 5 and 6) revealed that, in all exon contexts, U1comp effectively rescued the splicing pattern for all variants, with the highest correction impact on c.6429 + 5G > T (from 11 to 72%) in exon 22. Conversely, ExSpeU1s resulted in exon-dependent correction. In particular, ExSpeU1s were able to significantly rescue the splicing pattern for almost all variants in exon 6 and exon 22, with c.787 + 3 A > G (from 0 to 67%), c.787 + 5G > A (from 2–91%)(Fig. 4) and c.6429 + 5G > T (from 11–98%)(Fig. 6) being associated with the highest correction efficiency. In the exon 11 context (Fig. 5), while U1sh13 efficiently rescued splicing for all variants, the U1sh4 efficiently rescued the c.1752 + 5G > C (from 13 to 61%) variant, but not the c.1752 + 5G > T and c.1752 + 5G > A. The opposite was observed in the exon 22 context (Fig. 6), with U1sh6 able to efficiently rescue c.6429 + 5G > T (from 11 to 98%), but not U1sh15. Worthy, the variant c.787 + 2T > C was efficiently rescued by both compensatory and ExSpeU1, which is compatible with the observation that a small fraction of introns removed by the U2-type spliceosome has cytidine at position + 2 (Burset 2000) and strengthens the ability of engineered U1 to rescue + 2T > C variants, as confirmed by other studies (Scalet et al. 2019; Bar et al. 2017). Most importantly, for each exon context, we identified an ExSpeU1 that was more effective than the U1comp counterpart, a novel finding that further pushed forward the exploitation of the second-generation of U1snRNA variants. Although the molecular mechanism has not been deeply investigated, the specific gene context and splicing factors availability may contribute to the higher ExSpeU1snRNA efficiency.

## Conclusions

In this study we characterized a panel of 5’ss splicing changes located in three model *F8* exons and associated with different Hemophilia A phenotypes. The molecular characterization of splicing defects and their associated residual levels of correctly spliced transcripts, which were roughly consistent with the reported HA severity, elucidated the genotype-phenotype relationship. The lack of quantitative evaluation of splicing by current bioinformatic tools further highlights the importance of careful experimental investigations of molecular defects to assess pathogenicity, a key aspect of genetic diagnosis and counselling.

Significantly, for all changes within the same 5’ss, we identified a unique ExSpeU1 that outperformed the U1comp counterpart. This novel finding advances the use of second-generation U1snRNA variants to develop personalized RNA therapeutics designed to address exon-skipping variants, which are relatively common in human disease.

Future efforts should focus on further optimizing the design and delivery of these RNA-based therapeutics, as well as on evaluating their efficacy and safety in relevant disease models and clinical settings.

## Electronic supplementary material

Below is the link to the electronic supplementary material.

Supplementary Material 1

Supplementary Material 2

## Data Availability

All the data generated or analysed during this study are included in this published article and its additional files.

## Abbreviations

HA: Hemophilia A
U1snRNA: U1 small nuclear RNA
ExSpeU1: Exon Specific U1snRNA
FVIII: coagulation factor VIII
