# Supplementary material for: Rescue of a panel of Hemophilia A-causing 5’ss splicing mutations by unique Exon-specific U1snRNA variants
Source: Mol Med. 2025 Mar 27;31:121. doi: 10.1186/s10020-025-01176-8 (PMC11948882; doi:10.1186/s10020-025-01176-8)
Supplement: Supplementary file 1 — Supplementary Material 1 [file 10020_2025_1176_MOESM1_ESM.docx]

**Supplementary Table 1**

|  |  | **Splice Rover** | **MFE (kcal/mol)** | **MEM** | **MDD** | **MM** | **WMM** |
| --- | --- | --- | --- | --- | --- | --- | --- |
| F8IVS6 | **WT**  CAGgtatgt | 0,89 | -8.23 | 9,80 | 13,08 | 10,59 | 10,13 |
|  | **+2T>C**  CAGg**c**atgt | 0,00 | -4.74 | 2,05 | 5,32 | 2,84 | 2,38 |
|  | **+3A>T**  CAGgt**t**tgt | 0,63 | -7.29 | 7,44 | 11,18 | 6,6 | 5,18 |
|  | **+3A>G**  CAGgt**g**tgt | 0,70 | -7.85 | 8,08 | 11,68 | 7,20 | 5,75 |
|  | **+5G>A**  CAGgtat**a**t | 0,58 | -7.31 | 7,88 | 11,58 | 7,24 | 6,68 |
|  | **+6T>C**  CAGgtatg**c** | 0,75 | -7.31 | 9,37 | 12,48 | 9,03 | 8,90 |
| F8IVS11 | **WT**  CAGgtgagt | 0,99 | -11.94 | 10,64 | 14,88 | 11,86 | 11,69 |
|  | **+5G>A**  CAGgtga**a**t | 0,64 | -8.52 | 6,60 | 10,78 | 7,40 | 8,24 |
|  | **+5G>T**  CAGgtga**t**t | 0,64 | -8.35 | 5,95 | 9,98 | 6,84 | 7,79 |
|  | **+5G>C**  CAGgtga**c**t | 0,54 | -8.26 | 6,60 | 10,18 | 7 | 7,86 |
| F8IVS22 | **WT**  ATGgtatg | 0,99 | -5.20 | 8,35 | 11,98 | 7,95 | 7,72 |
|  | **+5G>T**  ATGgtat**t** | 0,78 | -4.28 | 3,85 | 6,28 | 3,97 | 3,82 |

Scores of the wild type or mutated 5’ss predicted by the SpliceRover, MaxEntScan and RNAcofold tools, with the available Maximum Entropy Model (MEM), Maximum Dependence Decomposition Model (MDD), First-order Markov Model (MM), Weight Matrix Model (WMM), and Minimum Free Energy (MFE), respectively. Figure 1A reports the mean ± standard deviation (SD) of all 5’ss score predictions after they are converted to percentages, with the value of the wild type set to 1 for each exon context. A brief explanation of each parameter is provided: Minimum Free Energy (MFE): Represents the thermodynamic stability of RNA secondary structures, predicted by RNAcofold. A lower MFE indicates a more stable structure. Maximum Entropy Model (MEM): Uses entropy-based probability distributions to predict splice site strength. It considers dependencies between positions in the sequence to improve accuracy. Maximum Dependence Decomposition Model (MDD): Analyzes dependencies between sequence positions to enhance splice site predictions. It builds decision trees based on sequence features. First-order Markov Model (MM): Assumes that the probability of a nucleotide depends only on the previous nucleotide. It's useful for capturing local sequence dependencies. Weight Matrix Model (WMM): Assigns weights to nucleotides at each position based on observed frequencies. It represents a simple probabilistic model for scoring sequences.
