# Supplementary material for: Rescue of a panel of Hemophilia A-causing 5’ss splicing mutations by unique Exon-specific U1snRNA variants
Source: Mol Med. 2025 Mar 27;31:121. doi: 10.1186/s10020-025-01176-8 (PMC11948882; doi:10.1186/s10020-025-01176-8)
Supplement: Supplementary file 2 — Supplementary Material 2 [file 10020_2025_1176_MOESM2_ESM.docx]

**Supplementary Table 2**

| **Primers name** | **Sequence 5’🡪3’** |
| --- | --- |
| Bra2 R | gtcaccaggaagttggttaaatca |
| Alfa globin F | caacttcaagctcctaagccactgc |
| **Mutagenesis** |  |
| F8 IVS11 +5 F | aaccaggtgacttcttgcctttc |
| F8 IVS11 +5 T F | aaccaggtgatttcttgcctttc |
| F8 IVS11 +5 A F | aaccaggtgaattcttgcctttc |
| F8 IVS11 +5 R | tcctctttgatctacagattc |
| F8 ex6 +2T>C F | gtctctgccaggcatgtacacac |
| F8 ex6 +3A>G F | gtctctgccaggtgtgtacacacc |
| F8 ex6 +3A>T F | gtctctgccaggtttgtacacacc |
| F8 ex6 +5G>A F | gtctctgccaggtatatacacacctg |
| F8 IVS6 mut R | ctgtttacataaccattgac |
| F8 ex22 +5G>T F | gaaccttaatggtatttaattagtcatttaaagg |
| F8 IVS22 mut R | cagtggaatttcctcgataag |
| **Golden Gate** |  |
| pTB G-G F | ggctaccgtctctttccaactgcccagcccgctagatatctcacatg |
| pTB G-G R | ggctaccgtctcatactgcacgcggtggtggctgtttag |
| F8 IVS11 G-G F | ggctaccgtctcaagtagtgaggaagagcttagaatgaat |
| F8 IVS11 G-G R | ggctaccgtctctggaaatcagctagagagagctaaatcc |
| F8 int. 5 (GG) F | ggctaccgtctcaagtagtggggatagggtttcaccatg |
| F8 int. 6 (GG) R | ggctaccgtctctggaacctattttaaaactggaaggc |
| F8 int. 21 (GG) F | ggctaccgtctcaagtactgacagtgatgttggggtg |
| F8 int. 22 (GG) R | ggctaccgtctctggaagctcacctctagagaattca |
| **U1 Primers** |  |
| U1 c R | atagaatacaagcttgcatgcctg |
| F8 IVS11 U1 comp. F | aggcccaagatctcatactcacctggcaggggagataccatgatca |
| U1ex11 sh4 F | aggcccaagatctcatgcaagaagtgcaggggagataccatgatca |
| U1ex11 sh13 F | aggcccaagatctcatcttggaaaggcaggggagataccatgatca |
| F8 IVS6 U1 comp. F | aggcccaagatctcatacatacctggcaggggagataccatgatca |
| U1 IVS6 sh7 F | aggcccaagatctcatcaggtgtgtgcaggggagataccatgatca |
| U1ex6 sh16 F | aggcccaagatctcatattgttgaggcaggggagataccatgatca |
| F8 IVS22 U1 comp. F | aggcccaagatctcatacataccatgcaggggagataccatgatca |
| U1 IVS22 sh6 F | aggcccaagatctcatgactaattagcaggggagataccatgatca |
| U1ex22 sh15 F | aggcccaagatctcatcctttaaatgcaggggagataccatgatca |

List of all primers exploited in the study.
